# TELEPSYCHOLOGY WITH ITS INNOVATIONS IN PSYCHOTHERAPY AND PSYCHODIAGNOSTICS

**DOI:** 10.1192/j.eurpsy.2023.1817

**Published:** 2023-07-19

**Authors:** I. S. Lancia, G. M. Festa, M. L. Sisinna, M. Ciarrocchi

**Affiliations:** 1 Interdisciplinary Institute of Higher Clinical Education (IAFeC); 2 Pontifical Faculty of Educational Sciences «AUXILIUM», Rome, Italy

## Abstract

**Introduction:**

The types of Electronic-Based Therapy & Intervention are spreading rapidly, especially after the onset of COVID, and several authors are dealing specifically with digital psychology and psychiatry, implementing new techniques and methodologies (Festa, Martinotti, 2022).The latest technological innovations related to metaverse and holography are emerging as new digital mediums and a reflection on their potential in the psychological field is urgent. Telepsychology, which currently uses text, audio and video-based communication as a medium, will see the way to relate to the patient with the “new digital presence” amplified at 360 degrees. This will of course revolutionize some aspects of classic psychological intervention: “physical proximity” will have a completely different meaning. New technologies through the digital medium have the power to cancel space, thus allowing the intervention to be separated from the sharing of the same physical environment. The first great epochal upheaval produced by new technologies in the field of the psychological profession is precisely this: psychological intervention no longer necessarily coincides with the sharing of the same physical space, space and intervention becoming independent.

**Objectives:**

We will soon and increasingly refer to associations, national and international professional associations and institutions that deal with the subject, such as the American Psychological Association, the International Society for Mental Health Online, the Online Therapy Institute, to have guidelines on best practice with respect to psychological performance online. This will be accompanied by specific training courses for clinicians who decide to work using technological tools and courses related to telepsychology and the development of a professional online identity.

**Methods:**

Holographic technology will revolutionize our “internal and external world”, and it is the task of psychological science to cultivate questions and provide answers both on the impact that this technology will have on the life of the average man and on the possibilities of use for our profession. The distance of the real world from the digital one is getting smaller with each passing day.

**Results:**

Our habits, our way of working, having fun have changed in different ways and in a very short time. Our physical, cognitive and sensory boundaries have expanded and our way of life has “evolved”.

**Conclusions:**

We find ourselves passing through and going through a phase, which began in the 1980s, which is not without shadows and settles into mass cultural aspects. The contradictions, abnormal uses and abuses of technology, but which accompany every innovative transformation and which we should gradually manage wisely, are also typical of this phase. We will see what the future holds!

**Disclosure of Interest:**

None Declared

